# Metabolic Stress and Compromised Identity of Pancreatic Beta Cells

**DOI:** 10.3389/fgene.2017.00021

**Published:** 2017-02-21

**Authors:** Avital Swisa, Benjamin Glaser, Yuval Dor

**Affiliations:** ^1^Department of Developmental Biology and Cancer Research, The Institute for Medical Research Israel-Canada, The Hebrew University-Hadassah Medical SchoolJerusalem, Israel; ^2^Endocrinology and Metabolism Service, Department of Internal Medicine, Hadassah-Hebrew University Medical CenterJerusalem, Israel

**Keywords:** Pax6, dedifferentiation, reprogramming, beta cell failure, type 2 diabetes mellitus, ghrelin, gastrin, oxidative stress

## Abstract

Beta cell failure is a central feature of type 2 diabetes (T2D), but the molecular underpinnings of the process remain only partly understood. It has been suggested that beta cell failure in T2D involves massive cell death. Other studies ascribe beta cell failure to cell exhaustion, due to chronic oxidative or endoplasmic reticulum stress leading to cellular dysfunction. More recently it was proposed that beta cells in T2D may lose their differentiated identity, possibly even gaining features of other islet cell types. The loss of beta cell identity appears to be driven by glucotoxicity inhibiting the activity of key beta cell transcription factors including Pdx1, Nkx6.1, MafA and Pax6, thereby silencing beta cell genes and derepressing alternative islet cell genes. The loss of beta cell identity is at least partly reversible upon normalization of glycemia, with implications for the reversibility of T2D, although it is not known if beta cell failure reaches eventually a point of no return. In this review we discuss current evidence for metabolism-driven compromised beta cell identity, key knowledge gaps and opportunities for utility in the treatment of T2D.

## Introduction

**FIGURE 1:**
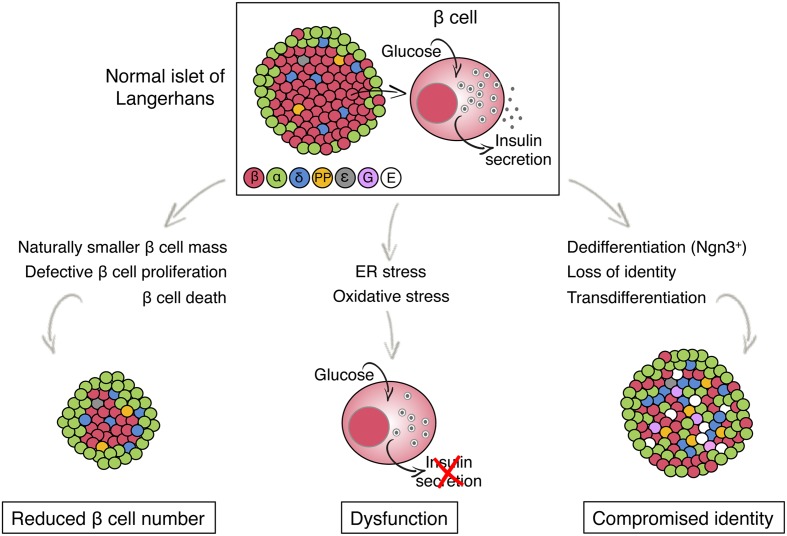
**Models for beta cell failure in T2D**. The illustration shows the architecture and endocrine cell composition of a normal pancreatic islet of Langerhans (top) and potential changes, distinguished by beta cell fate, that lead to beta cell failure in T2D (bottom). Different colors indicate different islet cell type. ε, ghrelin; G, gastrin, E, endocrine cell with empty granules (no hormone is produced).

The pathogenesis of T2D is thought to begin with lifestyle-induced insulin resistance, which gradually increases the demand on beta cells to secrete insulin. In the majority of people with this condition, increased beta cell function successfully maintains normoglycemia. In about 20% of insulin resistant individuals, beta cells eventually fail to deliver a sufficient amount of insulin, resulting in the development of glucose intolerance and ultimately fasting hyperglycemia and overt diabetes (Mokdad et al., 2001; Narayan et al., 2007; Alejandro et al., 2015). The genetic, cellular, and molecular basis for this failure of beta-cell compensation for increased demand has been studied intensively, but remains incompletely understood. Multiple theories have been proposed, ranging from low post natal beta-cell mass due to genetic or environmental (intrauterine) factors, to beta-cell factors resulting in differential susceptibility to metabolic stressors such as chronic hyperglycemia. The possible mechanisms leading to beta-cell failure can be broadly divided into three, not mutually exclusive categories as follows (summarized in **Figure 1**).

## Categories of Beta Cell Failure

### Reduced Beta Cell Numbers

Histological analysis of beta cell mass in autopsies of people with T2D has revealed that T2D patients have up to 50% decrease in beta cell mass compared with healthy individuals of the same BMI (Clark et al., 1988; Butler et al., 2003; Yoon et al., 2003; Rahier et al., 2008). This observation, combined with a qualitative histological demonstration of beta cell death, raised the possibility that beta cell failure involves beta cell death, resulting in reduced beta cell mass that cannot deliver sufficient insulin (Butler et al., 2003). Alternatively, it is possible that individuals with a lower potential for beta cell regeneration fail to increase beta cell mass when demand is higher, opening a gap between demand and supply that eventually causes hyperglycemia. It is also possible that individuals with genetically or environmentally determined low beta cell mass are prone to T2D, due to a compromised capacity for beta cell compensation upon increased demand later in life (Van Assche et al., 1977; Garofano et al., 1997; Petrik et al., 1999; Meier, 2009). The latter idea is consistent with a large variation in beta cell mass among healthy individuals, as observed in autopsies (Meier et al., 2008). In the absence of tools to assess the dynamics of beta cell mass in a given individual over time, it remains difficult to distinguish between these possibilities. However a common feature is that beta cell failure results, at least in part, from an irreversible deficiency in beta cell numbers. We note though that the modest reduction in beta cell mass is unlikely the “whole story,” since surgical removal of 50% of the functional beta-cell mass is not associated with glucose intolerance, at least not in the short-term (Kendall et al., 1990; Seaquist et al., 1996; Robertson et al., 2002).

### Beta Cell Dysfunction

A chronic load on beta cells may lead to severe dysfunction, such that histologically normal beta cells fail to secrete insulin properly in response to glucose. It has been proposed that metabolic load may lead to severe endoplasmic reticulum (ER) stress in beta cells, and that cellular adaptation to ER stress- namely the unfolded protein response (UPR)- can reduce glucose-stimulated insulin secretion (Izumi et al., 2003;Back and Kaufman, 2012; Evans-Molina et al., 2013). Alternatively, chronic oxidative stress resulting from improper glucose metabolism may cause beta cell dysfunction (Kaneto et al., 1999, 2002; Krauss et al., 2003; Robertson et al., 2003, 2007; Poitout and Robertson, 2008). Beta cells appear to be particularly sensitive to oxidative stress, as they express low levels of genes responsible for inactivation of reactive oxygen species (ROS). ROS are normally produced through mitochondrial metabolism, which is central in glucose-stimulated insulin secretion, and in normal conditions play a role in signaling pathways in beta cells. It was proposed that excessive ROS, generated as a consequence of persistent high glucose metabolism, suppress beta cell mitochondrial activity and other components of the insulin secretion pathway, thus leading to beta cell dysfunction (Lenzen, 2008; Drews et al., 2010). A characteristic of chronic beta cell stress is a situation termed “beta cell exhaustion,” where seemingly normal beta cells (judged by histological analysis, potentially including immunostaining for beta cell markers or even expression analysis) fail to secrete insulin properly. Per our current understanding of the phenomenon, beta cell exhaustion should be perfectly reversible upon normalization of glycemia. Indeed, short term (2–3 weeks) of intensive insulin treatment results in improved beta-cell function in patients with new-onset or long-standing T2D (Turner et al., 1976; Garvey et al., 1985; Glaser et al., 1988; Ilkova et al., 1997; Li et al., 2004; Ryan et al., 2004). The mechanism of improved beta-cell function is not fully understood, although improved proinsulin processing has been suggested (Glaser et al., 1988). That being said, although the positive effect on beta-cell function may be clinically significant, it is partial and in many cases transient, suggesting that reversible, functional exhaustion *per se* does not entirely explain beta-cell dysfunction seen in T2D. Other attempts in this context involve forcing “beta cell rest,” for example via temporary pharmacologic prevention of membrane depolarization, calcium entry, and insulin secretion (Greenwood et al., 1976; Guldstrand et al., 2002; Yoshikawa et al., 2004). Despite some positive initial reports, such approaches have not yielded a consistent improvement of beta cell function, potentially because of the interference with key signaling pathways within the beta cells. Furthermore, inhibition of beta-cell membrane depolarization may prevent the normal compensatory response to increased glycemic load (Porat et al., 2011).

### Loss of Beta Cell Identity

The beta-cell can be defined on a purely functional level as a cell capable of synthesizing, processing and secreting mature insulin in response to metabolic, hormonal and neurologic stimuli, or on a molecular level as a cell that expresses the full complement of genes associated with normal, regulated insulin secretion. In this review, we use the former definition to define “beta-cell function/dysfunction” as discussed above, and the latter definition to define “beta-cell identity.” Thus, for the purpose of this review, we define the loss of beta-cell identity as the failure to express the full complement of beta-cell genes or expression of genes not normally expressed in a mature healthy beta-cell.

Recently, a landmark study from the Accili group has described a mechanism for profound loss of beta cell function in diabetes, not involving cell death. Based on studies in mice with Foxo1-deficient beta cells they suggested that high metabolic load may perturb beta cell identity, via a process involving loss of the beta cell gene expression program, reversal to a fetal state (dedifferentiation), and reprogramming to express hormones of other islet cell types including glucagon and somatostatin (Talchai et al., 2012). Indeed, in mouse models of T2D, Talchai et al. (2012) found that the endocrine cell mass is maintained, revealed by immunostaining of chromogranin A and synaptophysin, despite massive loss of insulin, Pdx1 and MafA (termed “empty beta cells”). Recent studies have lent support to this reprograming model, including evidence for loss of beta cell identity in human T2D, although the extent of the phenomenon and its relevance for pathology remain unclear (Guo et al., 2013; White et al., 2013; Spijker et al., 2015; Brereton et al., 2016; Cinti et al., 2016). These studies have also shown that the phenomenon is largely reversible, such that dedifferentiated/reprogrammed beta cells appear to revert to their original identity when exposed to normal glucose levels (Laybutt et al., 2007; Blum et al., 2014; Brereton et al., 2014; Wang et al., 2014). It remains unclear whether the loss or change of beta cell phenotype becomes irreversible at some point. The latter is a crucial point, with implications to the feasibility of restoring beta cell mass in patients with T2D.

**FIGURE 2:**
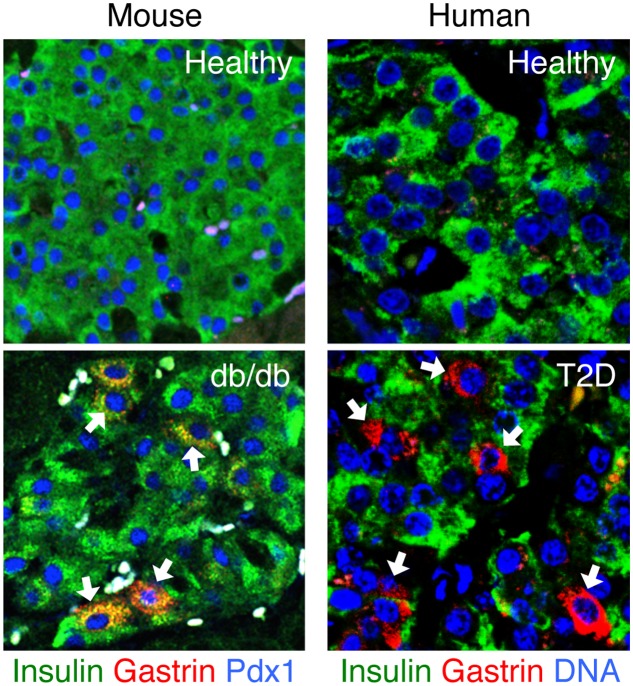
**Gastrin expression in islets of mice and humans with type 2 diabetes**. Gastrin expression in the pancreas is normally limited to fetal life. Islets of healthy adult mice and humans normally do not express gastrin. Strikingly, gastrin-positive cells re-appear in islets of mice and humans with type 2 diabetes. In diabetic db/db mice, gastrin is induced in beta cells; in humans with T2D, gastrin is induced mostly in delta cells. Sections are immunostained for gastrin (red), insulin (green), and Pdx1 or Dapi (blue). White arrows point to the gastrin positive cells. Micrographs are from our recently published paper (Dahan et al., 2017).

Recent work by our own group has contributed the observation that beta cells in human and rodent T2D may turn on expression of gastrin, a hormone typically expressed in the pancreas only during embryonic development and in rare islet cell tumors (Suissa et al., 2013; Dahan et al., 2017). While the physiological significance of gastrin expression remains unclear, we were able to use it as a biomarker of compromised identity and obtain insights into the dynamics and determinants of the process (see below). Gastrin expression is induced in beta cells upon exposure to high levels of glucose; importantly, gastrin expression does not involve the fetal endocrine progenitor marker and determinant neurogenin-3 (NeuroG3), which was proposed to mediate beta cell dedifferentiation (Talchai et al., 2012; Brereton et al., 2014; Wang et al., 2014). NeuroG3 mRNA and protein were not detected in islets of diabetic db/db mice that express gastrin, and gastrin expression in beta cells of diabetic mice occurred even when the NeuroG3 gene was deleted. We also showed that gastrin expression is reversible upon normalization of glycemia. Interestingly, while gastrin expression in diabetic mice occurred almost entirely in beta cells, islets of humans with T2D had gastrin expression in both beta and delta cells, with the latter accounting for most T2D-related islet gastrin expression. These findings suggest that metabolic stress causes the expression of gastrin in beta cells without going through a fetal-like stage. Therefore, the term “dedifferentiation” might be inappropriate, and the phenomenon is better described as reversible loss, partial or complete, of beta cell identity. **Figure 2** shows a visual example of islets in diabetic mice and humans that express gastrin.

## Molecular Mechanisms Linking Metabolic Stress to Loss of Beta Cell Identity

Emerging evidence from multiple lines of research is suggesting how metabolic stress, in particular hyperglycemia, may lead to a compromised cellular identity of beta cells.

### Role of Transcription Factors

As might have been expected, metabolic stress appears to converge on perturbation of the activity of transcription factors, the central regulators of cell identity and fate. We have extensive knowledge on the roles of many transcription factors in beta cell development, based on gene deletion experiments (reviewed in Mastracci and Sussel, 2012). However, most of these studies have disrupted the gene of interest starting in early developmental stages, using Insulin-Cre mice that delete the gene as early as insulin expression starts. Consequently, it is difficult to distinguish between a requirement for a particular gene in beta cell differentiation/maturation, and a role in the maintenance of mature beta cell function and identity. More recently, a number of studies used the more precise control over gene deletion afforded by Insulin-CreER mice, to induce gene deletion in adult beta cells using tamoxifen injection to activate Cre. These studies have revealed a requirement for multiple transcription factors in adult beta cells, and have established a paradigm where the maintenance of adult beta cell identity requires the continuous activity of many transcription factors. For example, deletion of Pdx1 in adult beta cells leads to loss of beta cell-specific genes, and to the induction of alpha cell genes, most prominently glucagon (Gao et al., 2014). Thus continuous activity of Pdx1 in adult beta cells is required not only for expression of beta cell genes but also for the prevention of expression of alpha cell genes. Interestingly, glucagon expression following Pdx1 deletion in beta cells is not stable; after 30 days, the proportion of these glucagon-expressing cells decreased from 35% to less than 15%. This indicates that loss of Pdx1 leads to a metastable state, which eventually shifts to other, more stable and yet to be defined states. An additional example is Nkx6.1, previously known to be important for beta cell differentiation during embryonic development (Sander et al., 2000). Deletion of Nkx6.1 in adult beta cells led to loss of beta cell-specific genes and functions, and over time caused the appearance of delta cell features including somatostatin expression (Taylor et al., 2013). MafA was also shown to be important for maintaining the transcriptional program of adult beta cells, and to prevent expression of disallowed genes (Nishimura et al., 2015).

**FIGURE 3:**
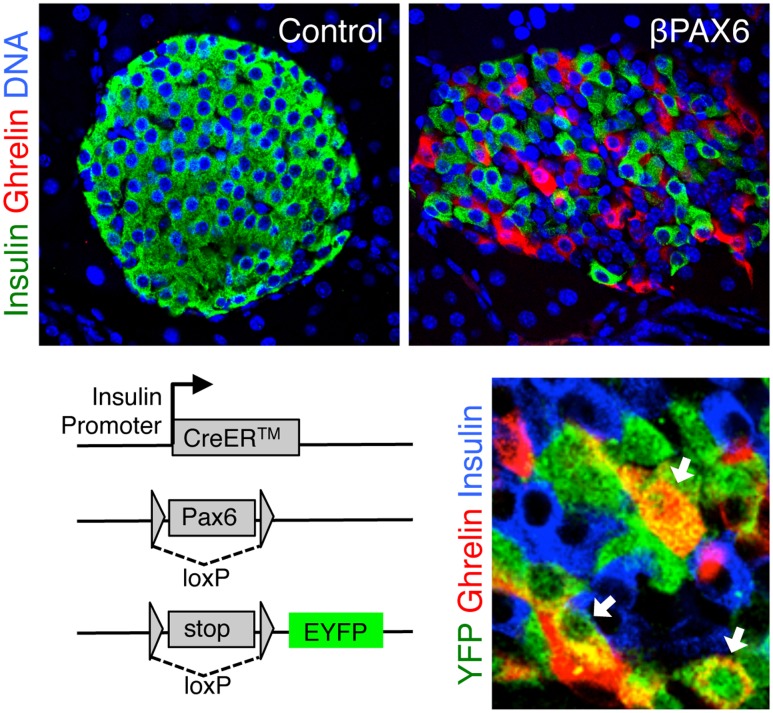
**Ghrelin expression following Pax6 loss in adult beta cells**. Top, pancreatic islets with ghrelin expression following Pax6 deletion in adult beta cells. Bottom left, deletion of pax6 was combined with Cre-mediated permanent expression of a yellow fluorescent protein (YFP) reporter. Bottom right, ghrelin-expressing cells do not express insulin, yet expression of YFP proves that these were insulin+ beta cells prior to the deletion of Pax6. Micrographs were adjusted from our published paper (Swisa et al., 2017).

Recently, we have shown that Pax6, another transcription factor previously implicated in developmental decisions, is essential for the maintenance of beta cell identity (Swisa et al., 2017). Deletion of Pax6 in adult beta cells led to a gradual loss of insulin expression, and to the induction of multiple genes characteristic of other islet cell types, including glucagon and somatostatin, and most prominently the fetal hormone ghrelin (**Figure 3**). Similarly, Nkx2.2 was found to be essential for the maintenance of adult beta cell identity, as its deletion led to down-regulation of beta cell genes and to the induction of genes typical of alternative islet cell types (Gutierrez et al., 2017). The main hormone induced in Nkx2.2-deficient beta cells was somatostatin, similarly to the phenotype of Nkx6.1-deficient beta cells (Schaffer et al., 2013). This may relate to Nkx2.2 being a transcriptional activator of Nkx6.1 (Sussel et al., 1998; Watada et al., 2000). Finally, the transcriptional co-activator LIM-Domain-Binding 1 (Ldb1), which is expressed preferentially in adult islets, and its binding partner Isl1, are essential for maintaining the expression of beta cell-specific genes by ensuring proper enhancer-promoter looping in the beta cell genome (Ediger et al., 2017).

Importantly for the concept of metabolic regulation of beta cell identity, many of the transcription factors mentioned above, specifically Pdx1, Nkx6.1, MafA and Pax6, as well as Foxo1, are inactivated by hyperglycemia (Jonas et al., 1999; Talchai et al., 2012; Guo et al., 2013; Brereton et al., 2014; Wang et al., 2014; Cinti et al., 2016), providing a plausible mechanism for compromised beta cell identity in diabetes. Notably, the factors appear to respond to chronic, rather than acute, high glucose levels; this suggests that their loss may not be the primary driver of beta cell failure, but rather a contributor to a vicious cycle of glucotoxicity.

### Direct Repression of Alternative Islet Cell Genes

**FIGURE 4:**
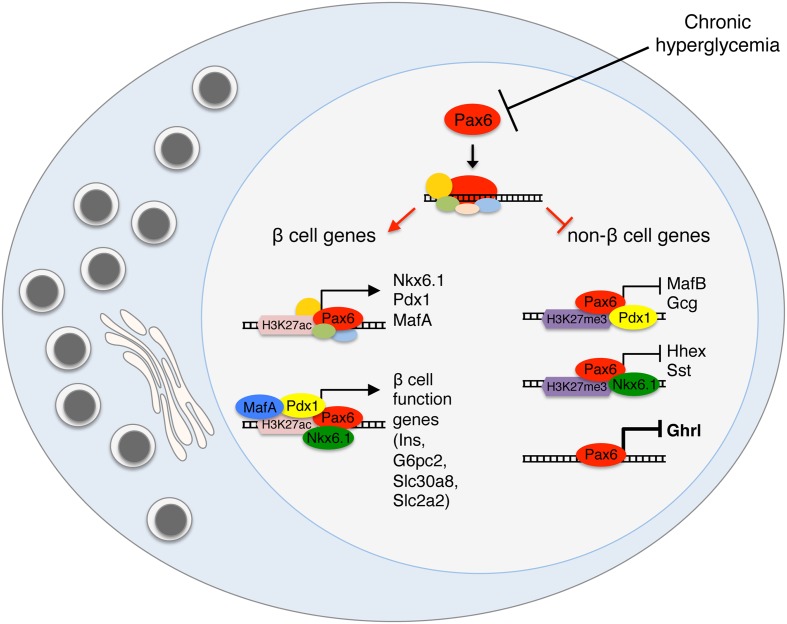
**Transcriptional activation and repression in the regulation of beta cell identity**. A model presenting the dual role of beta cell transcription factors as direct activators of beta cell genes and repressors of alternative islet cell genes. Pax6 activates the expression of key beta cell transcription factors such as Nkx6.1, Pdx1, and MafA and also interacts with these factors at the protein level, to execute the specific transcriptional outcome, activation or repression. We propose that the chromatin state and the other factors interacting in the transcriptional complex will determine the transcriptional activity.

How do transcription factors maintain beta cell identity? The obvious mechanism is via direct transcriptional activation of beta cell genes. This would explain reduced expression of beta cell genes following deletion of such transcription factors. However, the induction of non-beta cell genes following deletion of beta cell transcription factors is more difficult to explain. Interestingly, some well-established activators of beta cell genes have recently been found to act also as direct repressors of non-beta cell genes. This understanding is based on the integration of CHIP-Seq data on binding sites of transcription factors, together with data on genes up-regulated following transcription factor deletion. This integration exposes genes that are considered direct repression targets for a transcription factor. For example, Pdx1 binds and represses MafB transcription, helping to prevent an alpha cell program in beta cells (Gao et al., 2014). Rfx6, mostly known as an activator of beta cell maturation and function genes, is also a direct repressor of some “disallowed” beta cell genes such as LdhA (Pullen et al., 2010; Piccand et al., 2014). Nkx6.1 was also found to directly bind and repress a large group of genes, including the transcription factors Mnx1 and Rfx6 and the hormone somatostatin (Taylor et al., 2013). While studying how Pax6 controls beta cell identity, we discovered that it acts primarily as a direct repressor of gene expression in beta cells (Swisa et al., 2017). Hence in the absence (or inactivity) of Pax6, the transcription of beta cell specific genes is reduced, while the transcription of non-beta cell genes, such as alternative hormone genes, is derepressed. These studies collectively suggest that direct repression of non-beta cell genes is a central function of many beta cell transcription factors. A particularly important observation is that the regulatory effects of some beta cell transcription factors changes from the developmental stages to adults. For example, Nkx6.1 is necessary to repress Arx expression in Ngn^+^ embryonic endocrine progenitors, but it does not regulate Arx in differentiated beta cells (Schaffer et al., 2013). Interestingly, Nkx6.1 is still bound to Arx promoter in adult beta cells (Taylor et al., 2013) but does not execute any regulatory effect. How exactly do beta cell transcription factors act simultaneously as activators and repressors in the same cell, is an important open question. **Figure 4** presents a simplified model for the transcriptional regulation of beta cell identity, based on integration of our work with Pax6 deficient beta cells and studies of other transcription factors. We propose that Pax6 normally binds and activates genes of beta cell transcription factors and in addition, interacts with these factors to activate other beta cell genes or to repress non-beta cell genes. The transcriptional output (activation or repression) will depend on the factors participating in the transcriptional complex. Further investigation of the physical interactions and transcriptional hierarchy between Pax6 and more beta cell transcription factors (Arda et al., 2013) may shed light on transcriptional control mechanisms in beta cells and their link to metabolism.

### Signaling from Metabolic Stress to Loss of Transcription Factor Activity

What are the molecular mechanisms responsible for metabolic inactivation of key beta cell transcription factors? An elegant study from the Stein group (Guo et al., 2013) has shown that oxidative stress inhibits the activity of Pdx1, Nkx6.1, and MafA. Oxidative stress (mimicked *in vitro* by the addition of hydrogen peroxide) appears to inactivate these factors via at least two mechanisms: their activity as transcriptional activators is reduced, and in addition they are translocated from the nucleus to the cytoplasm where they are rapidly degraded. While the exact nature of the effect of oxidative stress on these factors remains to be elucidated, these findings provide a framework to understand how metabolic stress, manifested as increased oxidative stress and ROS, may impact gene expression in beta cells, including sensitive nodes controlling cell identity.

In our own work with Pax6, a different mechanism appears to be acting, as we have observed a significant down regulation of Pax6 at the mRNA level in beta cells of hyperglycemic mice (Swisa et al., 2017). The molecular mechanisms responsible for downregulation of Pax6 transcription in diabetes remain to be elucidated. We also note that in this case, relevance to the situation in human diabetes is not yet proven; we have observed downregualtion of Pax6 in diabetic db/db mice but not in islets from humans with T2D.

### Involvement of Membrane Depolarization and Calcium Signaling in Loss of Beta Cell Identity

Using gastrin expression as a biomarker for compromised beta cell identity, we explored how high levels of glucose signal to gastrin expression (Dahan et al., 2017). Surprisingly, we found that components of the classic triggering pathway for insulin secretion participate in the process. In the triggering pathway, ATP generated by oxidative metabolism causes the closure of ATP-dependent potassium channels (K_ATP_ channels); the resulting depolarization of the beta cell membrane leads to calcium entry, and the release of insulin granules. By preventing the closure of K_ATP_ channel, pharmacologically (using diazoxide) or genetically (using transgenic mice expressing a mutant version of the K_ATP_ channel Kir6.2), we showed that glucose-induced gastrin expression requires membrane depolarization. Furthermore, we found that the calcineurin inhibitor tacrolimus prevents glucose-induced gastrin expression; however, forced membrane depolarization or forced calcium entry under low glucose levels did not trigger gastrin expression. The conclusion from these experiments was that hyperglycemia triggers gastrin expression in beta cells via a pathway that includes mitochondrial metabolism, membrane depolarization, calcium entry and calcineurin activity; while membrane depolarization and calcium entry are necessary for gastrin expression, they are not sufficient. We speculate that the loss of beta cell identity requires also the direct action of ROS derived from aberrant mitochondrial metabolism, consistent with the Stein model emphasizing the importance of oxidative stress.

**FIGURE 5:**
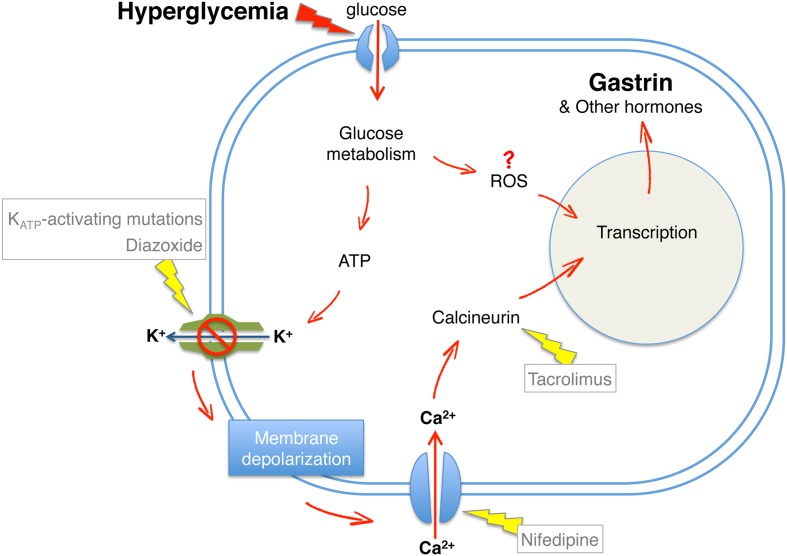
**Hyperglycemia-induced loss of beta cell identity**. A model summarizing downstream components of glucose signaling required for beta cell expression of gastrin following hyperglycemia. Red arrows, induction of beta cell reprograming; Yellow arrows, prevention of beta cell reprograming demonstrated by genetic or pharmacological interventions.

We note however that other studies, which have used different biomarkers of compromised beta cell identity (e.g., the appearance of cells co-expressing insulin and glucagon) in a model of K_ATP_-channel mutant mice, concluded that glucose triggers loss of beta cell identity regardless of membrane depolarization (Brereton et al., 2014; Wang et al., 2014). It is possible that different “illegitimate” programs of gene expression in beta cells require different combinations of signaling events. **Figure 5** shows a graphic summary of signaling pathways shown to mediate the effects of hyperglycemia on loss of beta cell identity.

## Fragile Identity of Beta Cells vs. Islet Cell Plasticity

The evidence presented above concerns conditions of metabolic stress, which compromise the identity of beta cells. Interestingly, independent lines of research indicate that islet cell identity can also be changed in the other direction, that is to drive non-beta cells to express beta cell genes. This was most strikingly demonstrated in studies from the Herrera group, which used adult mice in which the vast majority of beta cells were ablated using diphtheria toxin. This resulted in lethal diabetes, which required insulin treatment. Strikingly, it was found that a proportion of alpha cells in these mice underwent a conversion of identity to become insulin-producing beta cells (Thorel et al., 2010). When beta cells where ablated in young mice, a similar phenomenon of beta cell regeneration was observed, but this time the origins were somatostatin-expressing delta cells (Chera et al., 2014). Alpha cell reprogramming to beta cells in adult mice appeared not to require cell proliferation and to be NeuroG3-independent. In contrast, delta cell reprogramming to beta cells in newborn mice was found to involve dedifferentiation, expression of NeuroG3, proliferation and differentiation. Additional studies from the Collombat group found that genetic manipulations of the transcription factors Pax4 and Arx in adult alpha cells can also drive their conversion into functional insulin producing cells. Ectopic expression of Pax4, which is essential for beta cell differentiation from endocrine progenitors (Sosa-Pineda et al., 1997), was found to trigger insulin expression in alpha cells (Al-Hasani et al., 2013). It was suggested that Arx downregulation triggers this conversion as Arx deletion in alpha cells was sufficient to mediate alpha to beta cell reprograming, independently of Pax4 (Courtney et al., 2013). Strikingly, induced GABA signaling in alpha cells was recently shown to repress Arx and upregulate Pax4 in mouse and human alpha cells, leading to their reprograming to beta cells (Ben-Othman et al., 2017; Li et al., 2017), suggesting a pharmacological path to acquisition of beta cell identity.

**FIGURE 6:**
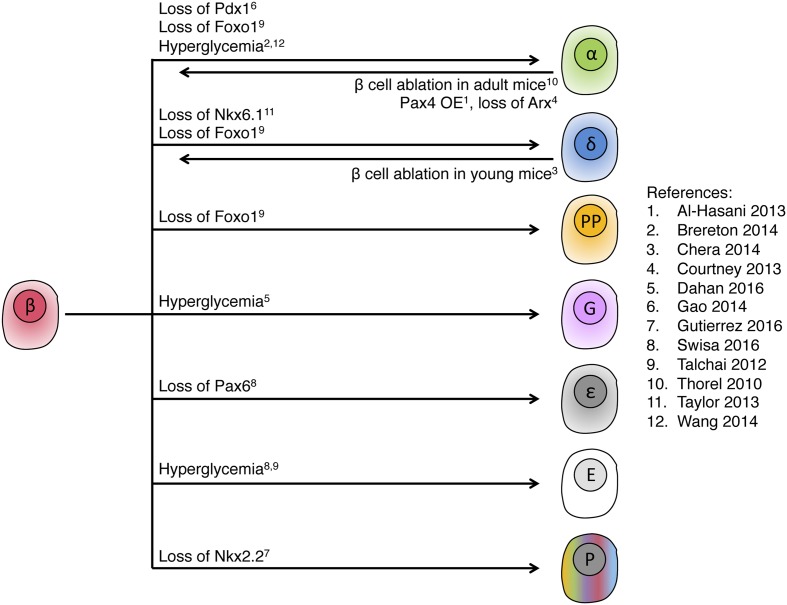
**Intra-islet cell identity transitions**. Summary of genetic manipulations that were shown to lead to intra-islet cell identity transitions in adult mice, indicating high plasticity within the pancreatic endocrine lineage. Note the fetal or abnormal islet cell types, ε, ghrelin; G, gastrin; P, polyhormonal; E, endocrine with empty granule. In most of the examples, only dominant transitions are presented. References cited in this figure: (1) Al-Hasani et al., 2013; (2) Brereton et al., 2014; (3) Chera et al., 2014; (4) Courtney et al., 2013; (5) Dahan et al., 2017; (6) Gao et al., 2014; (7) Gutierrez et al., 2017; (8) Swisa et al., 2017; (9) Talchai et al., 2012; (10) Thorel et al., 2010; (11) Taylor et al., 2013; (12) Wang et al., 2014.

When combined with evidence for loss of beta cell identity in diabetes, a picture emerges of a rather fragile identity of islet cells, where metabolic insults compromise cell identity. A striking common feature to all these models is the retention of islet cell identity: stressed beta cells in diabetes may lose beta cell markers and gain markers of alpha, delta, ghrelin or gastrin cells, but they never convert to non-islet cells such as hepatocytes, for example. Similarly, the formation of beta cells from non-beta cells appears to occur mostly, if not solely, from other islet cells and not from more distant lineages. **Figure 6** summarizes current evidence of bidirectional islet cell identity flow, from and to beta cells.

Thus, islet endocrine identity appears to be robust while islet cell type identity is fragile and dependent on metabolic state. What might be the molecular basis for this peculiar situation? A clue comes from comparisons of the transcriptome and epigenome of distinct islet cell types, obtained recently from FACS-sorted beta and alpha cells (Bramswig et al., 2013; Wang et al., 2016). These studies have revealed that different islet cell types are extremely similar both in their expression profile and epigenetic makeup (patterns of histone modifications and DNA methylome). The deep epigenetic similarity between different islet cell types may explain intra-islet cell plasticity, from and to beta cells, and the difficulty in generating islet cells from non-islet tissue.

## Summary and Perspective

Compromised beta cell identity is emerging as an important contributor to beta cell failure in diabetes. Most evidence so far suggests that the phenomenon results from hyperglycemia-induced inactivation of key transcription factors, acting as activators of gene expression in beta cells as well as repressors of non non-beta cell genes. Beta cells with a compromised identity become dysfunctional and fail to secrete insulin in response to glucose. They may show just loss of beta cell genes, but also a gain of non-beta cell genes. Importantly, a generic endocrine identity remains in such cells (e.g., they do not reprogram to cells of the exocrine pancreas or other non-islet cell types), and sometimes they even acquire expression of genes typical to other islet cell types. The loss of beta cell identity seen in diabetes, and even the acquisition of non-beta cell identity, appear to be reversible upon normalization of metabolism. Islet cell plasticity, while contributing to beta cell failure in diabetes, may also work in the opposite direction and be utilized to generate new beta cells from other islet cell types. The concept of metabolic compromise of beta cell identity opens up many fascinating questions, with implications for our understanding of tissue dynamics and for approaches to prevent and cure diabetes. Some key open questions in this field include:

• *Reversibility of changes*. The loss of beta cell identity and the acquisition of features of non-beta cell genes appear to be reversible upon normalization of glucose levels. It remains unclear whether at some point the acquisition of alternate islet cell identity becomes stable, and independent of metabolic state. This is important for loss of beta cell identity under stress, but also for reprogramming of other cell types to beta cells.
• *The involvement of NeuroG3 and fetal islet programs in changes of islet cell identity.* Several papers reported expression of NeuroG3 in adult beta cells upon loss of key beta cell transcription factors (Talchai et al., 2012; Taylor et al., 2013), as well as in delta cells when they convert to beta cells after extreme beta cell ablation (Chera et al., 2014). However it remains unclear if NeuroG3 is required for these changes of fate. At least the expression of gastrin in beta cells is NeuroG3-independent (Dahan et al., 2017).
• *The direction of identity changes.* Metabolic insults were reported to result in beta cell neogenesis, and at the same time loss of beta cell identity. It will be essential to understand the determinants of cell fate transitions within islets.
• *The relative importance of compromised beta cell identity to beta cell failure in diabetes*, compared with beta cell death and beta cell dysfunction.

Answers to these questions, which will surely emerge in coming years, may suggest novel directions to prevent or reverse beta cell failure in diabetes.

## Author Contributions

AS, BG, and YD wrote together the manuscript.

## Conflict of Interest Statement

The authors declare that the research was conducted in the absence of any commercial or financial relationships that could be construed as a potential conflict of interest. The reviewer IL and handling Editor declared their shared affiliation, and the handling Editor states that the process nevertheless met the standards of a fair and objective review.
